# Atrioventricular Nodal Re-entry Tachycardia in Identical Twins: A Case Report and Literature Review

**DOI:** 10.1016/s0972-6292(16)30589-7

**Published:** 2013-01-01

**Authors:** Walid Barake, Jane Caldwell, Adrian Baranchuk

**Affiliations:** Heart Rhythm Service, Queen's University, Kingston, Ontario, Canada

**Keywords:** Twins, Paroxysmal supraventricular tachycardia, AVNRT

## Abstract

This report details the case of 17 year old identical twins who both presented with paroxysmal supraventricular tachycardia (PSVT). Electrophysiological studies revealed atrioventricular nodal reentry tachycardia (AVNRT) in both twins. Successful but technically challenging slow pathway ablation was performed in both twins. This is the first reported case of confirmed AVNRT in identical twins which adds strong evidence to heritability of the dual AV node physiology and AVNRT. A review of the current literature regarding PSVT in monozygotic twins is provided.

## Introduction

Paroxysmal supraventricular tachycardia (PSVT) caused by accessory pathways or dual atrioventricular node (AVN) physiology were initially thought to be randomly occurring congenital anomalies.[1,2] However, familial clustering of AVNRT has previously been described [1], as has the incidental discovery of dual AVN pathways in identical twins. [3] This report is the first documentation of identical twins with AVNRT suggesting a genetic influence [1] rather than a random congenital error of cardiogenesis.

## Case Report

### History and examination

Identical female twins were referred with palpitations from a young age. Twin A was first seen in clinic at the age of 13 years. She had been experiencing rapid palpitations since the age of 12. Her palpitations were triggered by activity and occurred roughly x 4-5/month. The episodes were associated with breathlessness and presyncope. Most episodes were terminated with vagal maneuvers with only one episode required intravenous adenosine. Physical exam and echocardiography of twin A were unremarkable. Her resting 12 lead ECG was normal bar left axis deviation. During tachycardia, the12-lead ECG during palpitations showed a narrow complex tachycardia (rate = 180 bpm) with retrograde negative P waves in lead II about 20 ms off the onset of the QRS. Oral metoprolol (25 mg twice daily) reduced the frequency of the episodes but did not abolish them and hence the decision for management with catheter ablation.

Twin B was first seen in clinic at the age of 11 years of age. She had experienced palpitations once every 2-3 months since the age of 10. Again the episodes were related to exertion and were associated with shortness of breath and presyncope. The episodes were terminated by lying flat. Examination, echocardiography and resting 12-lead ECG were normal bar left axis deviation. On this occasion, SVT was diagnosed by an external event recorder. Initial management was with vagal maneuvers only until her palpitations became more frequent at the age of 17 when more invasive management was sought.

### Electrophysiological study

A standard 4 wire study was performed in both patients: decapolar CSL response to coronary sinus from the left subclavian vein, johnson curve quadrapolar catheters to high right atrial and right ventricular apical positions, and a 4 mm non-irrigated tip ablation catheter initially in the His position (St Jude's Medical, St Paul's, MN, USA).

**Figure 1:**
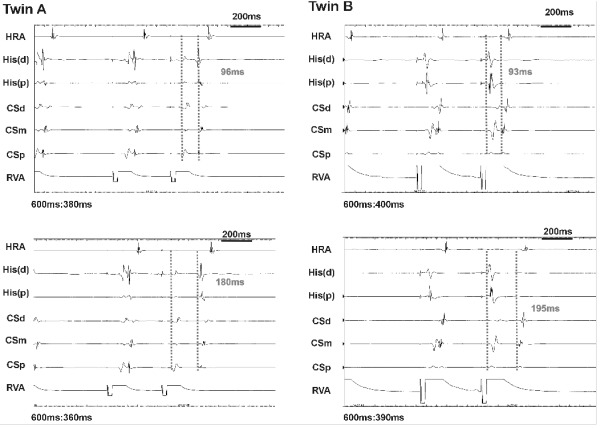
The four panels show intracardiac tracings during ventricular pacing. A jump in VA conduction time was observed in both twins with similar jumps at similar pacing intervals i.e. a VA jump of 84ms from S2=380ms to S2=360ms (drive chain 600ms) in twin A, and a jump of 102ms from S2=400ms to S2=390ms in twin B (drive chain 600ms).

*Twin A:* Basic intervals were within normal limits (Table 1). Retrograde properties were assessed at pacing cycle length of 600 ms and 400 ms. At both cycle lengths, retrograde activation was concentric and decremental but with demonstrable dual nodal physiology as seen by a VA jump (Figure 1). At 600 ms, the fast pathway effective refractory period (FPERP) was 360 ms, and slow pathway ERP (SPERP) 320 ms. At pacing cycle length of 400 ms, FPERP was 330 ms and SPERP 290 ms. Assessment of the antegrade properties was performed at pacing cycle length of 600 ms. Again dual pathway physiology was demonstrated with FPERP 300 ms and SPERP of less than 210 ms. The antegrade Wenckebach cycle length was less than 240 ms. Tachycardia was easily induced with the administration of 1 mcg/min of Isoproterenol.

**Figure 2:**
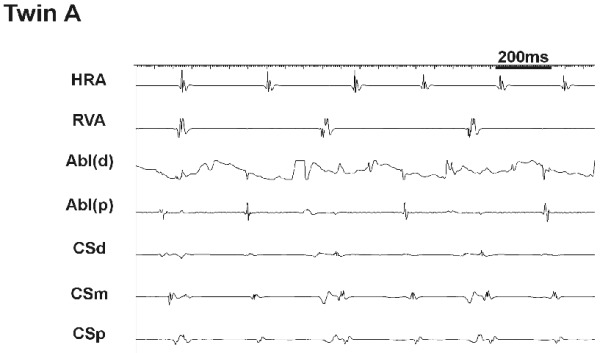
Intracardiac recordings during RF application at the slow pathway region in twin A. Fast junctional tachycardia with dissociated atrial and ventricular activity was observed leading to the curtailment of the RF application.

Although successful slow pathway ablation was achieved this was a technically challenging affair requiring a total of 34 radiofrequency (RF) applications. Many of the RF applications had to be curtailed due to fast junctional tachycardia with dissociated atrial and ventricular activity (Figure 2). After this salvo of burns, AVNRT could no longer be induced. Post ablation, the AV nodal parameters were marginally altered as expected; antegrade Wenckebach cycle length 270ms, AH-89 ms, HV-38 ms and QRS-65 ms. During the subsequent 2 years follow-up no clinical recurrence of AVNRT was observed.

**Figure 3:**
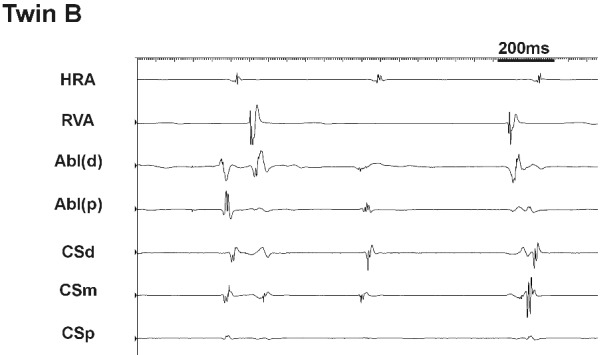
Intracardiac recordings during mapping at the slow pathway region in twin B. Junctional tachycardia with dissociated atrial and ventricular activity was observed on touch alone.

**Figure 4:**
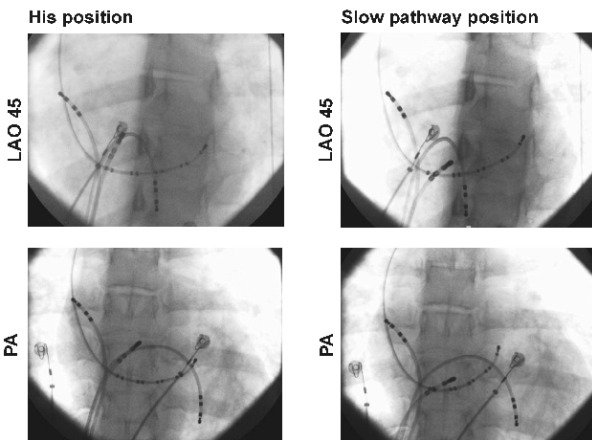
Fluoroscopy during ablation of twin B. Left panel: the ablation catheter is at the point of maximal His recording. Right panel: the catheter is in the position where touch AV block and junctional tachycardia were produced.

*Twin B:* Baseline intervals were normal (Table 1). Retrograde testing showed a decremental, concentric VA conduction with AV node ERP of 240 msec. Antegrade conduction was decremental with FPERP 390ms and SPERP 240ms on a 600 ms train. Isoprenaline was infused at 2 mcg/min and AVNRT was induced by burst pacing. We therefore proceeded to slow pathway ablation. Successful slow pathway ablation was obtained after 9 RF applications and the procedure was fraught with difficulties; both transient AV block and junctional tachycardia were produced by touching the slow pathway area (Figure 3) even though this was distant from the maximal His recording (Figure 4).

## Discussion

Paroxysmal supraventricular tachycardia is the term used to describe intermittent SVTs other than atrial fibrillation, atrial flutter and multifocal atrial tachycardia (AT); i.e. a re-entrant origin. PSVT occurs with an incidence of 35 per 100,000 people a year [1].

The major causes of PSVTs are AVNRT (~60%), and AV re-entrant tachycardia (AVRT) (~ 30 %) with smaller contributions from intra-atrial re-entry, micro re-entrant AT and sinus nodal re-entry [5].

PSVT caused by AV accessory pathways or dual AVN physiology were formerly thought to be randomly occurring congenital anomalies due to pathological substrates present from birth.[1,2] However, several studies based on familial clustering and twins case studies suggest a substantial genetic influence in the pathophysiology of PSVT. The case presented here is the first to report AVNRT in identical twins.

Back in 1976, Mispireta et al [6] were the first to report PSVT in twins, describing pre-excitation syndrome in monozygotic twin brothers. Both brothers presented with palpitations. Both patients demonstrated pre-excitation; one of them was found to have WPW and episodes of PSVTs were documented, some of these with antegrade conduction through the normal pathway, and others with conduction through the anomalous pathway. The other twin had Lown-Ganong-Levine (L-G-L) syndrome, demonstrated by electrocardiography and vectorcardiography. They suggested that these pre-excitation syndromes might have a sex linked genetic basis [6].

A similar clinical scenario was reported by Bennett et al [11]. Here identical 10 year old male twins with identical surface electrocardiograms both experienced arrhythmia. One twin experienced episodes of rapid palpitations and on one occasion was resuscitated from ventricular fibrillation. Electrophysiological (EP) study confirmed the presence of both LGL (AVN bypass tract) and WPW pathways. The other twin was asymptomatic and was found to have LGL syndrome by EP study without any additional AV accessory pathways.

In more recent studies investigating familial clustering of pre-excitation syndromes, an autosomal dominant pattern of inheritance has been suggested [7-9]. Even more recently, studies have identified missense mutations in the PRKAG2 gene in some cases of familial WPW especially associated with an early onset of atrial fibrillation and conduction disease [8,9]. PRKAG2 is the gene encoding for the gamma-2 regulatory subunit of cAMP-activated protein kinase. A mutation (Arg531Gly) in the gamma-2 regulatory subunit (PRKAG2) of AMP-activated protein kinase (AMPK) was found to be responsible for the syndrome. These observations confirm an important functional role of AMPK in the regulation of ion channels specific to cardiac tissue [9]. PRKAG2 mutation has been also associated with the development of nodoventricular accessory pathways (Mahaim fibers) [10].

The possibility of inheritable AVNRT has been suggested by a couple of case reports. Lu et al demonstrated bystander dual AVN physiology in 16-year-old identical female twins who both have AVRT caused by the same left lateral AV accessory pathway [3].

In the case presented here, the presence of dual nodal physiology with symptomatic AVNRT, similar retrograde jumps in VA conduction (Figure 1) and similar technical difficulties during RF ablation (Figure 2 and 3) in identical twins suggest an inheritable component to AV node development. The challenging ablation could not be explained simply by a close location of the ablation catheter to the AV node as demonstrated radiologically in twin B (Figure 4). Potential explanations for the phenomena are (i) inferior extension of the AV node to the level of the mouth of the coronary sinus [12] or (ii) arterial blood supply of the AV node running near the CS ostium [13].

These 2 instances could justify the presence of AV dissociation during manipulation of the catheters in the slow pathway region or application of RF in the same area.

## Conclusion

The unique case presented here adds evidence to the inheritability of AVNRT as previously suggested. Further studies of AVNRT involving genetic mapping in correlation with the delineation of the AV nodal arterial supply and histological presence of inferior nodal extensions may be warranted.

## Figures and Tables

**Table 1:**
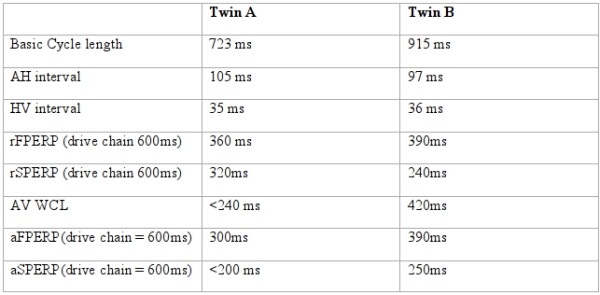
Electrophysiological parameters for both twins

AH = Atrio-His; HV = His-Ventricular; rFPERP = retrograde fast pathway effective refractory period of AV node; rSPERP = retrograde Slow Pathway effective refractory period; AV WCL = Atrioventricular Wenckebach cycle length; aFPERP = antegrade fast pathway effective refractory period of AV node; aSPERP = antegrade Slow Pathway effective refractory period of the AV node.
